# Who is asked about alcohol consumption? A retrospective cohort study using a national repository of Electronic Medical Records

**DOI:** 10.1016/j.pmedr.2021.101346

**Published:** 2021-03-09

**Authors:** Alexander Singer, Leanne Kosowan, Shilpa Loewen, Sheryl Spitoff, Michelle Greiver, Joanna Lynch

**Affiliations:** a Max Rady College of Medicine, Rady Faculty of Health Sciences, University of Manitoba, Winnipeg, Manitoba, Canada; b Department of Family and Community Medicine, University of Toronto, Toronto, Ontario, Canada

**Keywords:** EMR, Electronic Medical Record, CPCSSN, Canadian Primary Care Sentential Surveillance Network, OR, Odds Ratio, CI, Confidence Interval, Electronic health records, Alcohol drinking, Primary health care, Supervised machine learning

## Abstract

Documentation of alcohol use in electronic medical record (EMR) informs interventions to reduce alcohol-related morbidity and mortality. This retrospective cohort study explored EMR data from 960 primary care providers participating in the Canadian Primary Care Sentinel Surveillance Network to describe documentation of alcohol use (e.g. none, current or past use) in the EMR. Included providers represented 700,620 adult patients from across Canada with an encounter between 2015 and 2018. Bivariate comparisons characterized the patients with, and without, documentation of alcohol use. Multivariate generalized estimating equation models with logit function assessed patient and provider characteristics associated with (1) documentation of alcohol and (2) patients with heightened risk for alcohol-related problems. Forty percent of patients had alcohol use documentation in the EMR. Light alcohol consumption was recorded for 43.6% of these patients. Male patients (OR1.09, CI 1.07–1.12), who were older (OR1.26, CI 1.23–1.30), had more frequent visits to their provider (OR1.11, CI 1.09–1.13) and had hypertension (OR1.07, CI 1.06–1.09) or depression (OR1.07, CI 1.09–1.14) had higher odds of alcohol documentation. There were 4.7% of patients with a record indicating heightened risk for alcohol-related problems. Male patients (OR3.27 CI 3.14–3.4), patients with depression (OR2.01 CI1.93–2.1) and rural residency (OR1.35 CI1.29–1.42) was associated with risk for alcohol-related problems. Heavy alcohol consumption is associated with an increased risk of negative health outcomes, particularly for patients with certain chronic conditions. However, these patients do not have alcohol use consistently documented in the EMR. Strategies should be designed and implemented to support more consistent alcohol-screening among high-risk patients.

## 1 Introduction

Heavy consumption of alcohol is a significant cause of preventable death and is associated with a variety of comorbidities, including liver disease, cardiovascular disease, mental health conditions and malignancy. Globally, alcohol resulted in 5.3% of all mortality in 2016 (Shield et al., 2012, World Health Organization, 2018). The Canadian Centre on Substance Use and Addiction established low-risk drinking guidelines in 2011, which were aimed at the reduction of health risks and negative effects of alcohol consumption (Butt et al., 2011). The low-risk drinking guidelines advise males to not consume more than 15 standard drinks per week or 3 drinks per day, and females to not consume more than 10 standard drinks per week or 2 drinks per day. However, in 2019 15% of Canadians who consume alcohol exceed the established low-risk drinking guidelines (Butt et al., 2011, Canadian Centre on Substance Use and Addition, 2019). In 2019, Myran et al reported that the number of emergency visits attributable to alcohol use increased greater than the overall increase in emergency visits (Myran et al., 2019). There is evidence that supports screening for alcohol use and brief interventions for heavy alcohol use as effective for the reduction of heavy alcohol consumption (Zur and Zaric, 2016, Kaner et al., 2018, O’Donnell et al., 2014, Spithoff and Kahan, 2015a, Spithoff and Kahan, 2015b). In a meta-analysis, Kaner et al. found that brief interventions in primary care reduced alcohol consumption at 12 months (Zur and Zaric, 2016). However, evidence to support their effectiveness in sub-populations, and the optimum length and frequency to maintain long-term effectiveness remain unknown (Kaner et al., 2018).

In 2015, a study of primary care patients from six European countries found that general practitioners were more likely to identify smoking (88.4%) than alcohol use (64.6%) among patients that self-reported use. (Manthey et al., 2015) In 2015, a study evaluating documented tobacco use within Canadian Electronic Medical Records (EMR) found that 64.4% of patients had available information (Greiver et al., 2015). In contrast, a study considering EMRs from Alberta, Canada found that in 2013 only 20% of patient records had documentation regarding alcohol use (Mitchell et al., 2012). A meta-analysis by Mitchell *et al*. in 2012 showed that primary care physicians identified alcohol use disorders among 41.7% (95% CI 23.0–61.7) of their patients, but only 27.3% (95% CI 16.9–39.1) of the patients had documentation of alcohol use in their primary care record (Mitchell et al., 2012). Sub-optimal documentation of alcohol use can limit a provider’s ability to intervene and reduce alcohol related morbidity and mortality (Torti et al., 2013, Rehm et al., 2016). The frequency that alcohol use is documented overall in Canadian primary care settings is unknown.

Similarly, although research has evaluated trends in documentation of other important risk factors such as tobacco use (Greiver et al., 2015) there is limited research available to describe trends including patient and provider characteristics associated with documentation of alcohol use in the EMR. Research has shown that general practitioners rely on clinical judgement and consider patient self-reported health and social consequences prior to asking a patient about their alcohol use. (Manthey et al., 2015, Mitchell et al., 2012) To better understand documentation of alcohol use this study aimed to describe current rates of alcohol use documentation in primary care EMRs in Canada, as well as understand patient or provider characteristics associated with documentation.

## 2 Methods

We conducted a retrospective cohort study using primary care EMR data from the Canadian Primary Care Sentential Surveillance Network (CPCSSN) repository. The CPCSSN extracts, de-identifies and processes EMR derived information from 1574 primary care providers in 8 provinces across Canada. (Queenan et al., 2016). Participation in CPCSSN is available to all primary care family physicians, nurse practitioners and community pediatricians located in a jurisdiction with a local CPCSSN network. Participation varies between provinces and continues to increase. Using a combined manual and automated process the extracted EMR information is placed into multiple tables that make up the repository (e.g. patient demographics, provider and site specifics, billing, encounter diagnosis, health conditions, medication, laboratory results, exam, risk factor). Risk factors typically recorded in the EMR include alcohol, smoking, physical activity, nutrition and obesity. The purpose of CPCSSN is to facilitate surveillance, research and quality improvement in Canadian primary care settings. The patient population within CPCSSN has been shown to be representative of the general populations in Canada when compared to other national data sources. (Queenan et al., 2016) This study included demographic information of the patient and the provider (sex, age, postal code, provider type), chronic diseases with validated definitions (Williamson et al., 2014) in the CPCSSN repository (diabetes, hypertension, COPD, depression, osteoarthritis, epilepsy, Parkinson’s disease and dementia), visit frequency and alcohol documentation from the risk factor table.

### 2.1 Study population

Data were extracted from 960 providers across Canada. Adult patients (≥18 years of age) with at least one encounter to a participating CPCSSN primary care provider between January 1, 2015 and January 1, 2018 were included in the study. This study applied a supervised machine learning algorithm to process the unstructured text in the CPCSSN risk factor table. Algorithms were used to identify documentation of alcohol use and create groups based on the amount of alcohol consumed. Patients without documentation of alcohol use suggests that the provider did not ask the patient about their alcohol consumption or did not recorded their response in the EMR. Two reviewers conducted a manual chart review to validate the alcohol categories that were created. A third reviewer assessed the key words and phrases within each of the alcohol categories (Appendix A).

Alcohol use can be categorized into three groups: consumes alcohol, past drinker, or non-drinker. We created sub-groups for alcohol consumption based on the low-risk drinking guidelines (Butt et al., 2011, Canadian Centre on Substance Use and Addition, 2019). Alcohol consumption could be light (<3 drinks a week), moderate (3–15 drinks a week) or heavy (>15 drinks a week). After alcohol consumption amounts were categorized we used a supervised machine learning algorithm to categorize the remaining chats using key words and phrases identified during a chart review. Appendix A provides a complete list of the key words included within each group. The most common key words used to identify past alcohol consumption were ‘Alcoholics Anonymous’, ‘recovering’, ‘recovered’, and ‘past’. A small percentage of records, titled ‘unknown’, focused on family history, health conditions or did not specify the amount of alcohol consumed.

### 2.2 Covariates

Urban residency was defined as a patient with a postal code from a Canadian Metropolitan Area (population ≥ 100,000 people) (Statistics Canada, 2018). Previously validated case definitions were applied to capture diagnoses of diabetes, hypertension, COPD, depression, osteoarthritis, epilepsy, Parkinson’s disease and dementia (Williamson et al., 2014). Comorbidites diabetes, hypertension, depression and osteoarthritis were included within our multivariate regression due to their significance in previous literature (Rehm et al., 2017, Rehm et al., 2010, Rehm et al., 2017, Roerecke et al., 2017, Polsky and Akturk, 2017, Schrieks et al., 2015, Baliunas et al., 2009, Boden and Fergusson, 2011, Pavkovic et al., 2018, Sullivan et al., 2005, Patten et al., 2015) and within our chi-square analyses. Patient and provider age were dichotomized and represented as higher than the mean age (50.7 years and 49.6 years, respectively). Visit frequency was categorized into patients with an average (median) of ≤ 3 visits a year to their primary care provider compared to patients with an average (median) of > 3 visits a year (IQR 4).

### 2.3 Analysis

We characterized the patients with and without documentation of alcohol in the EMR using descriptive statistics including mean (standard deviation [SD]) and frequency. Bivariate comparisons assessed similarities and differences between patients who had, or did not have, documentation of alcohol in the EMR. Among patients with documentation of alcohol in the EMR we determined the prevalence of each consumption sub-group (i.e. never, light, moderate, heavy, and past) and compared patients with a heightened risk of alcohol-related problems (Heavy drinker, or past drinker) to patients that did not drink, or had light and moderate alcohol consumption.

We performed two multivariable logistic model with generalized estimating equations. The first assessed associations between documentation of alcohol in the risk factor table (yes vs. no) and patient factors (i.e. sex (male vs. female), age (>50.7 years vs. ≤ 50.7 Years), residency (rural vs urban), depression (yes vs no), diabetes (yes vs no), hypertension (yes vs no) osteoporosis (yes vs no), visit frequency (>3 annual visits vs ≤ 3 visit annually) and provider factors (i.e. sex (male vs. female), age (>49.6 years vs. ≤ 49.6 years)). The second regression included patients with documentation of alcohol to examine patients with heightened risk for alcohol-related problems (yes vs no) and patient factors (i.e. sex (male vs. female), age (continuous), residency (rural vs urban), depression (yes vs no), diabetes (yes vs no), hypertension (yes vs no) osteoporosis (yes vs no), visit frequency (>3 annual visits vs ≤ 3 visit annually). The GEE model considered repetition of provider to control for practice size of the provider within the model. Odds ratios (OR) and 95% confidence intervals (CI) are reported.

Statistical analysis was performed using SAS software 9.4. Copyright © [2002–2012] SAS Institute Inc. SAS and all other SAS Institute Inc. product or service names are registered trademarks or trademarks of SAS Institute Inc., Cary, NC, USA. Ethics approval for this study was obtained from the Health Research Ethics Board at the University of Manitoba.

## 3 Results

There were 700,620 adult patients who saw a CPCSSN participating provider between 2015 and 2018. The vast majority of these patients (99.4%) saw a family physician rather than a nurse practitioner. Less than half (40.6%) of patients had alcohol use documented in the EMR. The majority of alcohol use documentation referred to light consumption of alcohol (43.6%), followed by moderate intake (30.4%) and patients who do not consume alcohol (21.4%). Patients with past alcohol use (1.7%) or heavy consumption (3.0%) are at heightened risk for alcohol-related problems (Table 1).

**Table 1:** Documentations of Alcohol Use in the Electronic Medical Record of CPCSSN participating primary care providers.

Alcohol category	Percent (n)
Non-drinker	21.4% (57,712)
Light	43.6% (117,779)
Moderate	30.4% (82,178)
Heavy	3.0% (8088)
Past	1.7% (4519)
Total*	270,276

*There were 13,992 patients with documentation of alcohol in the EMR that were not classified (i.e. record focused on family history, health conditions or did not specific an amount).

Alcohol documentation was slightly more common among male patients compared to female patients (42.4% vs. 43.7%). Among patients with alcohol documentation male patients were significantly more likely to report heavy alcohol consumption compared to female patients (3.2% vs. 1.4%), whereas female patients were more likely to report light alcohol consumption compared to male patients (25.7% vs. 17.9%) (<0.0001). Patients with documented alcohol use in the EMR were also older on average (51.9 years, SD 16.7), than patients who did not have documentation (49.9 years, SD 18.2) (<0.0001). The average age of patients with heavy alcohol consumption was 55.3 years (SD15.6). Documentation of alcohol use in the EMR was also more common among patients that visited the primary care provider more frequently (5 visits annually (SD5.9) vs. 4.8 visits annually (SD5.8)) (<0.0001). Patients with comorbidities were more likely to have documentation in the EMR related to alcohol compared to those with no comorbidities (49.4% vs. 45.7%), particularly if they had a diagnosis of hypertension, depression, osteoarthritis, or diabetes (Table 2) (p < 0.0001). Similarly, patients that reported heavy alcohol consumption were more likely to have one or more of these referenced comorbidities in the EMR (63.5%), while patients with light or moderate alcohol consumption were more likely to have no comorbidities (54.5% vs. 51.4%, respectively) (<0.0001). Diagnoses of COPD, dementia, epilepsy, and Parkinson’s disease were not associated with increased rates of alcohol documentation or heavy alcohol consumption.

**Table 2:** Descriptive of patients in the Canadian Primary Care Sentinel Surveillance Network repository with, and without documentation of alcohol in the Electronic Medical Record between 2015 and 2018. N = 700,620

Variable name	Patients with no record for alcohol n = 416,352 (59.4%)	Patients with a record for alcohol n = 284,268 (40.6%)	P-Value
*Patient Characteristics*			
Male patient (vs. female patient) n (%, CI)	176,457 (42.4, 0.422–0.425)	124,243 (43.7, 0.435–0.439)	<0.0001
Patient age mean, ((SD) CI)	49.9 ((18.2), 49.8–50.0)	51.9 ((16.7), 51.8–52.0)	<0.0001
Urban Residence (vs. Rural Residency) n (%, CI)	340,473 (81.8, 0.817–0.819)	251,801 (88.6, 0.885–0.887)	<0.0001
≥1 *comorbidity (vs. no comorbidities) n (%, CI)	190,250 (45.7, 0.455–0.459)	140,552 (49.4, 0.493–0.496)	<0.0001
COPD n (%, CI)	16,260 (3.9, 0.039–0.04)	11308, (4.0, 0.039–0.041)	0.2808
Dementia n (%, CI)	6995 (1.7, 0.016–0.017)	4708 (1.7, 0.016–0.017)	0.4629
Hypertension n (%, CI)	94,472 (22.7, 0.226–0.228)	72,431 (25.5, 0.253–0.256)	<0.0001
Depression n (%, CI)	75,959 18.3 (0.181–0.184)	53,263 (18.7, 0.186–0.189)	<0.0001
Diabetes n (%, CI)	44,212 (10.6, 0.105–0.107)	33,322 (11.7, 0.116–0.118)	<0.0001
Epilepsy n (%, CI)	5084 (1.2, 0.012–0.013)	3421 (1.2, 0.012–0.012)	0.9635
Parkinson’s Disease n (%, CI)	1449 (0.4, 0.003–0.004)	1010 (0.4, 0.003–0.004)	0.6684
Osteoarthritis n (%, CI)	54,079 (13.0, 0.129–0.131)	41,923 (14.8, 0.146–0.149)	<0.0001
Visit Frequency mean, ((SD) CI)	4.8((5.6) 4.77–4.8)	5.0((5.9) 4.96–5.0)	<0.0001

*Provider Characteristics*			
Female provider (vs. male provider) n (%, CI)	193,729 (48.7, 0.486–0.489)	139,500 (51.9, 0.518–0.521)	<0.0001
Provider age mean, ((SD) CI)	50.5((10.9), 50.5–50.6)	48.3((11.3), 48.3–48.4)	<0.0001
Family Physician (vs. Nurse Practitioner) n (%, CI)	354,139 (99.4, 0.993–0.994)	246,050 (99.5, 0.994–0.995)	<0.0001

*Comorbidity is defined as one or more diagnosis in the EMR of COPD, Dementia, Hypertension, Depression, Diabetes, Epilepsy, Parkinson’s Disease, and/or Osteoarthritis.

**CI: 95% Confidence Interval.

Male patients had 1.09 times higher odds of having documentation of alcohol use in the EMR compared to female patients (CI 1.07–1.12). Older patients (OR1.26, CI 1.23–1.30) and patients who had > 3 visited annually to their primary care provider (OR1.11, CI 1.09–1.13) had higher odds of having documentation related to alcohol compared to younger patients and patients who had ≤ 3 visits annually (Table 3). Patients diagnosed with hypertension or depression were 1.07 times more likely to have documentation of alcohol use compared to patients without one of these diagnoses (CI 1.06–1.09; CI 1.09–1.14, respectively). When we controlled for patient and provider factors, a diagnosis of diabetes or osteoporosis were not associated with an increase in documentation of alcohol use in the EMR (Table 3).

**Table 3:** Multi-variate Generalized Estimate Equation Model with Logit Function of Factors Associated with a record of alcohol use in the Electronic Medical Records of patients who visited a Primary Care Provider Participating in CPCSSN. N = 700,620

Variable name	Unadjusted Odds Ratio (95% CI)	Adjusted Odds Ratio (95% CI)	p-value
Male patient (vs. female patient)	1.07 (1.06–1.81)	1.09 (1.07–1.12)	**<0.0001**
Patient age (>50.7 years vs. ≤50.7 Years)	1.19 (1.18–1.2)	1.26 (1.23–1.30)	**<0.0001**
Rural (vs. Urban)	0.56 (0.55–0.57)	1.00 (0.98–1.02)	0.6527
>3 visits annually (vs. ≤3 visit annually)	1.14 (1.13–1.15)	1.11 (1.09–1.13)	**<0.0001**
Depression (vs. no depression)	1.03 (1.02–1.04)	1.11 (1.09–1.14)	**<0.0001**
Diabetes (vs. no diabetes)	1.11 (1.09–1.13)	0.95 (0.93–0.98)	**0.0001**
Hypertension (vs. no hypertension)	1.17 (1.15–1.18)	1.07 (1.06–1.09)	**<0.0001**
Osteoporosis (vs. no osteoporosis)	1.16 (1.14–1.17)	1.01 (0.99–1.03)	0.1146
Male provider (vs. female provider)	0.88 (0.87–0.89)	0.90 (0.67–1.18)	0.4328
Provider age (>49.6 years vs. ≤ 49.6 years)	1.32 (1.30–1.33)	1.09 (0.81–1.46)	0.5906

*CI: Confidence Interval.

*CPCSSN: Canadian Primary Care Sentential Surveillance Network.

Patients with heightened risk for alcohol related problems were more likely than patients with never, light or moderate alcohol use to be male (OR 3.27, CI3.14–3.4), reside in a rural area (OR 1.34, CI 1.29–1.42) and to be diagnosed with depression (OR 2.01, CI 1.93–2.1) or hypertension (OR 1.24, CI 1.19–1.3) (Table 4).

**Table 4:** Multi-variate Generalized Estimate Equation Model with Logit Function of Factors Associated with a record of past or heavy alcohol consumption in the Electronic Medical Records of patients with documentation of alcohol use by a Primary Care Provider Participating in CPCSSN. N = 270276

Variable name	Unadjusted Odds Ratio (95% CI)	Adjusted Odds Ratio (95% CI)	p-value
Male patient (vs. female patient)	3.08 (2.96–3.2)	3.27 (3.14–3.4)	**<0.0001**
Patient age (per 1 year increase in age)	1.01 (1.01–1.01)	1.01 (1.01–1.01)	**<0.0001**
Rural (vs. Urban)	1.45 (1.37–1.52)	1.35 (1.29–1.42)	**<0.0001**
>3 visits annually (vs. ≤ 3 visit annually)	1.34 (1.26–1.42)	1.23 (1.15–1.32)	**<0.0001**
Depression (vs. no depression)	1.76 (1.69–1.83)	2.01 (1.93–2.1)	**<0.0001**
Diabetes (vs. no diabetes)	1.22 (1.15–1.28)	0.83 (0.78–0.88)	**0.0001**
Hypertension (vs. no hypertension)	1.6 (1.54–1.66)	1.24 (1.19–1.30)	**<0.0001**
Osteoporosis (vs. no osteoporosis)	1.28 (1.22–1.34)	1.04 (0.99–1.1)	0.1260

*CI: Confidence Interval.

*CPCSSN: Canadian Primary Care Sentential Surveillance Network.

## 4 Discussion

This study represents the single largest cohort of primary care provider EMR data in Canada, providing a nationally representative understanding of documentation of alcohol use in primary care. Less than half (40.6%) of patients in our cohort had documentation of alcohol use in the EMR. This is significantly lower than documentation of alcohol use in a European study (64.6%) (Manthey et al., 2015), but we found double the rate of EMR documentation previously reported in Alberta, Canada (20.0%) (Torti et al., 2013). The higher prevalence of alcohol screening compared to the study focused in Alberta, Canada could be related to this larger sample, an increase in knowledge of alcohol screening or an increase in EMR data quality with ongoing use. Alcohol documentation in the EMR frequently described light consumption of alcohol and infrequently mentioned heavy or past alcohol consumption. Compared to other literature (Butt et al., 2011; [4]) we found significantly less patients had documentation indicating alcohol consumption above the low-risk drinking guidelines (3% vs 15%).

This result indicates that documentation of alcohol use in the EMR is still lower than EMR documentation of tobacco use (Greiver et al., 2015). Current guidelines for alcohol screening in adults recommend asking all individuals 18 years or older about their use of alcohol (Butt et al., 2011, Canadian Centre on Substance Use and Addition, 2019, Shimizu et al., 2016, Curry et al., 2018). The Canadian Pediatric Society also suggest routine screening of alcohol use among patients aged 12–17, (Curry et al., 2018) due to the prevalence of problems associated with alcohol use during adolescents as well as the effectiveness of brief alcohol interventions in primary care for this age group (Greig et al., 2016, Newton et al., 2018).

Screening and documentation of alcohol use in the EMR is not without its challenges. Several studies have described potential barriers in screening for alcohol use, including patient reluctance to discuss substance use, clinical knowledge and training related to screening tools and interventions, and system factors (e.g. resources and time) (Manthey et al., 2015, Rehm et al., 2016, McNeely et al., 2018, Kim et al., 2013). Similar to the study describing documentation of tobacco use (Greiver et al., 2015), there was no association between provider characteristics and alcohol documentation. This suggests that targeting providers for interventions is unlikely to be successful. However, Greiver and colleges did find an association between length of EMR use and increased documentation. Providers that used the EMR for 4 years or longer had higher odds of tobacco documentation in the EMR (Greiver et al., 2015).

In this study, patients with documentation of alcohol use tended to be older, male patients who visited a primary care clinic >3 times a year. Male patients were also significantly more likely to have documentation indicating heightened risk for alcohol-related problems. The global prevalence of alcohol use disorders is five times more common in men than women (Williamson et al., 2014); providers aware of this may be more likely to screen male patients (Carvalho et al., 2019). However, Myran et al. reported that although men and lower-income individuals continue to experience the highest number of emergency department visits attributed to alcohol use, women and younger adults had a greater increase in the number of alcohol related emergency department visits between 2003 and 2016 (Myran et al., 2019). Interestingly alcohol related emergency department visits suggest a need for greater attention to these sub-populations. The findings suggest that younger patients may be under-screened due to their reduced interaction with the health care system and therefore, lack of opportunities to be screened. Approximately 34% of all alcohol related disability adjusted life years (DALYs) can be attributed to younger adults (aged 15–29 years) (Rehm et al., 2009). In contrast, the mean age of patients with documentation regarding alcohol use in this study was 51.9 years (SD 16.7) and among patients with documentation indicating heightened risk 55.3 years (SD 15.6) suggesting a need to target screening interventions to younger patients.

A growing body of research evidence suggests that patients with a comorbidity associated with alcohol consumption should be screened for alcohol use (Rehm et al., 2016; Roerecke et al., 2017). Similar to our study, other literature has found an association between heavy alcohol consumption and hypertension and cardiovascular disease (World Health Organization, 2018, Rehm et al., 2017, Rehm et al., 2010). A recent meta-analysis showed marked improvements in blood pressure with reduction in alcohol consumption to near abstinence for those who previously drank more than 2 drinks per day (Roerecke et al., 2017). There has been a push in the European Union to increase alcohol screening in newly diagnosed hypertensive patients (Rehm et al., 2017). While the odds of having alcohol use documented in hypertensive patients was higher than patients without a diagnosis, not all patients with hypertension in this study were screened for alcohol use. Additionally, patients with a heightened risk of alcohol-related problems also had increased odds of a hypertension diagnosis. The link between alcohol use and hypertension suggests these patients may benefit from increased alcohol screening and intervention.

Similarly, evidence has found an association between alcohol consumption and diabetes mellitus (Butt et al., 2011, Polsky and Akturk, 2017, Schrieks et al., 2015, Baliunas et al., 2009). Moderate alcohol consumption has been associated with increased insulin sensitivity and therefore a reduction in the risk of type 2 diabetes mellitus (Butt et al., 2011, Polsky and Akturk, 2017, Schrieks et al., 2015, Baliunas et al., 2009). However, chronic heavy alcohol use (≥5 drinks a day) may increase the risk of diabetes and its complications (Butt et al., 2011, Polsky and Akturk, 2017, Schrieks et al., 2015, Baliunas et al., 2009). In this study, a diagnosis of diabetes mellitus slightly reduced the odds of alcohol use documentation after controlling for covariates. Given the relationship between diabetes mellitus and alcohol use, further research to explore the relationship between alcohol use and diabetes mellitus, supported with documentation of alcohol use, can inform patient care practices.

Patients with depression were 11% more likely to have alcohol use documentation in the EMR and were significantly more likely to be categorized with heightened risk for alcohol-related problems. Since heavy alcohol consumption contributes to incidence and worsening of major depression (Boden and Fergusson, 2011, Pavkovic et al., 2018, Sullivan et al., 2005), it is not surprising that depression and documentation of alcohol use were associated. A Canadian study found that depressed patients were 19.5 (CI 15.9–23.1) times more likely to have co-occurring alcohol abuse problem compared to patients without depression (Patten et al., 2015). Mental health problems are often identified and managed within the primary care setting (Ferenchick et al., 2019); improving documentation of alcohol use can inform care for patients that may have co-occurring depression.

### 4.1 Limitations

While the observational design allows for consideration of a large national database, we can only report associations not causation using this design. Primary care providers participating in CPCSSN may not be representative of the overall make-up of Canadian or international primary care providers (Queenan et al., 2016). However, when adjusted for age and sex the patients represented in the CPCSSN repository are representative of the general population in Canada (Queenan et al., 2016). These results may not represent jurisdictions beyond Canadian primary care settings.

EMR data are entered by primary care providers for clinical use and hence, data quality may not be a central priority for providers. The CPCSSN extraction of risk factor data focuses on the structured and semi-structured fields in the EMR. The processing algorithms and unique EMR characteristics may have prevented retrieval of some alcohol use documentation. Alcohol use documented in the narrative notes or prenatal records may not be captured by CPCSSN. In 2013 Torti et al. reviewed documentation of alcohol use for one Canadian province using the CPCSSN dataset. They reported that 75% of alcohol use documentation occurred within the risk factor table14. Although we suspect we may be underrepresenting the overall capture rates of alcohol use documentation we expect that the data quality exercises at CPCSSN and EMR improvements have improved our capture rates. Although we performed an internal validation of the alcohol use categories we did not perform an external validation of these groups due to the lack of an available reference standard. Alcohol use represents documentation in the EMR and may not represent actual alcohol use of the patient. Documentation of alcohol use is not necessarily synonymous with appropriate screening for alcohol use disorder which this study did not assess.

## 5 Conclusion

This study highlighted the existing gaps in documentation of alcohol use among high-risk groups known to have a greater prevalence of alcohol use problems. Some groups did have slightly higher rates of alcohol use documentation such as male patients and those with hypertension and depression. Future quality improvement activities and research can focus on optimizing approaches for targeted screening in high-risk individuals, such as patients diagnosed with diabetes and younger patients, for alcohol use in primary care clinics.

## CRediT authorship contribution statement

**Alexander Singer:** Conceptualization, Methodology, Writing - review & editing, Supervision. **Leanne Kosowan:** Conceptualization, Methodology, Software, Validation, Formal analysis, Writing - review & editing. **Shilpa Loewen:** Writing - original draft. **Sheryl Spitoff:** Writing - review & editing. **Michelle Greiver:** Writing - review & editing. **Joanna Lynch:** Writing - review & editing.

## Declaration of Competing Interest

The authors declare that they have no known competing financial interests or personal relationships that could have appeared to influence the work reported in this paper.
